# Clinical Features and Surgical Outcomes of the Children With Urolithiasis at a Tertiary Care Hospital: First Report From Somalia

**DOI:** 10.3389/fped.2022.930136

**Published:** 2022-06-21

**Authors:** Aşir Eraslan, Abdikarim Hussein Mohamed, Sertac Cimen

**Affiliations:** Mogadishu Somalia Turkish Training and Research Hospital, Mogadishu, Somalia

**Keywords:** pediatric, urolithiasis, minimally invasive procedures, Somalia, Sub-Saharan Africa

## Abstract

**Introduction:**

There are very few studies regarding pediatric urolithiasis (PU) reported from Africa, and to date, no data have been presented from Somalia. This study evaluated the sociodemographic and radiological characteristics, treatment, and outcome data of the PU patients treated at Somalia's only tertiary care center.

**Method:**

The data of all patients diagnosed with urolithiasis during a 6-year period were reviewed. Only pediatric (age <18) urolithiasis patients were included. Demographic parameters, radiological features, stone characteristics, treatment, and outcome data were collected and analyzed.

**Results:**

Overall, 227 (127 male, 100 female) patients were included. The rate of PU was 8.1%. The mean patient age was 12.7 ± 3.2. More than two-thirds of the patients (*n* = 161, 70.9%) were adolescents. The stones were located in the kidney in 50.7%, the ureter in 33%, and the bladder in 14.5%. Distal ureteral stones (36%) accounted for the majority of ureteral stones, followed by the ureterovesical junction (26.7%) and proximal ureteral (24%) stones. The mean stone size was 16.2 mm. Most (42.3%) stones had a 10–20 mm diameter, while 23.3% were sized between 6 and 10 mm. Renal insufficiency was present in 5.3%. Among 227 patients, 101 (44.5%) underwent minimally invasive procedures including ureterorenoscopic lithotripsy (*n* = 40, 18%), retrograde intrarenal surgery (*n* = 30, 13.2%) and percutaneous nephrolithotomy (*n* = 31, 14%). Open pyelolithotomy was the most common surgery performed (*n* = 53, 22.3%). Surgical site infection developed following 3.5% of the open surgery cases. The stone-free rate was 91.3%. It was significantly higher in open cases (98%) compared to the cases performed via a minimally invasive approach (83%) (*p* = 0.02).

**Conclusion:**

In Somalia, PU is more common than in many other countries. Open surgery continues to be the primary treatment modality for children with urolithiasis due to the restricted endourology resources. However, minimally invasive approaches have evolved over the last years.

## Introduction

Pediatric urolithiasis (PU) is a rare condition accounting for 1–3% of all urolithiasis cases (1). Over the last decades, pediatric urinary stone disease incidence has increased progressively; however, its actual incidence remains unclear (2, 3). In addition, it was reported that the frequency of PU increased among African Americans and the female patient population (4).

Most PU cases were reported from developing countries such as India, Pakistan, and Turkey (1). As per previously published data, the rate of adult urolithiasis is 5% in Asia, 12% in Canada, 5–9% in Europe, and 13–15% in the United States (5–8).

Lifestyle and dietary practices, socioeconomic status, climate characteristics, and familial predisposition play an essential role in stone formation in the pediatric population (2, 9). In addition, up to 60% of children with stones have underlying metabolic disturbances, among which hypercalciuria and hypocitraturia are the most common (10). Additional risk factors include genitourinary anatomical abnormalities and recurrent UTIs (10, 11).

It was reported that PU has a 30–66% recurrence rate (12). The mean interval to stone recurrence is 3–6 years, and higher recurrence rates and increased risk of end-stage renal disease were documented in cases with metabolic abnormalities. In addition, the European Association of Urology (EAU) guidelines considered PU a high-risk factor for the recurrence of stone disease and the requirement of invasive interventions (6). Thus, PU is associated with increased morbidity and healthcare-related expenses.

Although it is known that the prevalence of PU varies significantly worldwide, to date, no reports have been published regarding PU in Somalia. Therefore, this study aimed to analyze PU patients' sociodemographic and radiological characteristics, treatment, and outcome data at a tertiary care center in Somalia.

## Method

Somalia is an African country with a total land area of 627,340 Km^2^. The current population of Somalia has been estimated at 16,618,919 persons, and the median age is 16.7 years based on Worldometer elaboration of the latest United Nations data. Fifty-four percent of the population lives in rural areas. Mogadishu Somali Turkish Training and Research Hospital is the only tertiary care center in the country. The hospital was established in the capital city of Mogadishu in 2015, offering various types of surgical procedures and imaging modalities, including ultrasound (US), computed tomography (CT), and magnetic resonance imaging (MRI).

Before commencing this study, approval was obtained from the institutional ethical review board of Mogadishu Somalia Turkish training and Research Hospital (MSTH-9006/21.02.2022). In addition, all study participants previously consented to the use of their medical and surgical data in the context of this study.

Data of the patients who received the diagnostic code of urolithiasis in agreement with the International Classification of Diseases (ICD-10) system between January 2016 and December 2021 were retrospectively reviewed. Pediatric (<18 years) patients diagnosed with urolithiasis and treated at our center constituted the target population of this study. However, those with incomplete data or those who did not consent to the study were excluded. The primary outcome measure was the prevalence and radiological characteristics of pediatric urolithiasis. The secondary outcome measure was the feasibility of minimally invasive approaches and alternative procedures in low-resource environments.

Data including demographic parameters (i.e., age and gender), presence or absence of azotemia, serum uric acid, calcium, phosphate levels, and urinalysis findings were retrieved from the electronic patient folders. In addition, data regarding urolithiasis characteristics, radiological features, medical treatments, and surgical procedures were collected.

The evaluated characteristics of urolithiasis were location and number of stones, laterality status (i.e., unilateral vs. bilateral) accompanying urologic abnormalities, and grade of hydronephrosis. Diagnosis of urolithiasis was based on the abdominal US, kidney-ureter-bladder (KUB) X-ray, and abdominopelvic non-contrast computed tomography (NCCT). In addition, because of the unavailability of renal scintigraphy, patients with severe cortical thinning or relatively higher grades of hydronephrosis were assessed using CT urography or intravenous urography to assess split renal functions. These data were also included in the patient files.

Surgical data collected for the study included the type of surgical intervention and postoperative outcomes. The patients were considered stone-free if there were no residual stones or in the presence of residual stone fragments ≤ 4 mm in size on X-ray KUB performed 1 month after surgery. In addition, patients were assessed regarding complications during a 1-month postoperative follow-up period.

The data were analyzed using univariate descriptive statistics. The categorical variables were presented as frequencies and percentages, while the quantitative variables were expressed as means ± standard deviations (SD). Cross-tabulations and the Chi-square test were used to determine the association between the variables. A *p*-value of < 0.05 was considered a statistical significance. Statistical analyses were performed using the Statistical Package for Social Sciences (SPSS v23, IBM, Armonk, NY, USA) software.

## Results

Our review revealed that 2,806 patients were diagnosed with urolithiasis during the study period. Among these, 227 were in the pediatric age group. Therefore, the rate of PU was 8.1%. All of these patients had complete data and consented to the study; thus, none were excluded. The mean age of the study population was 12.7 ± 3.2 [0.5–17] years.

Slight male predisposition was detected; 55.9% (*n* = 127) of all cases were male while 44.1% (*n* = 100) were female, with a male to female ratio of 1.3:1. All patients underwent US as the initial imaging modality. In addition, an NCCT was performed as an adjunct imaging method in 79.2% (*n* = 180) of the entire cohort.

**Figure 1:**
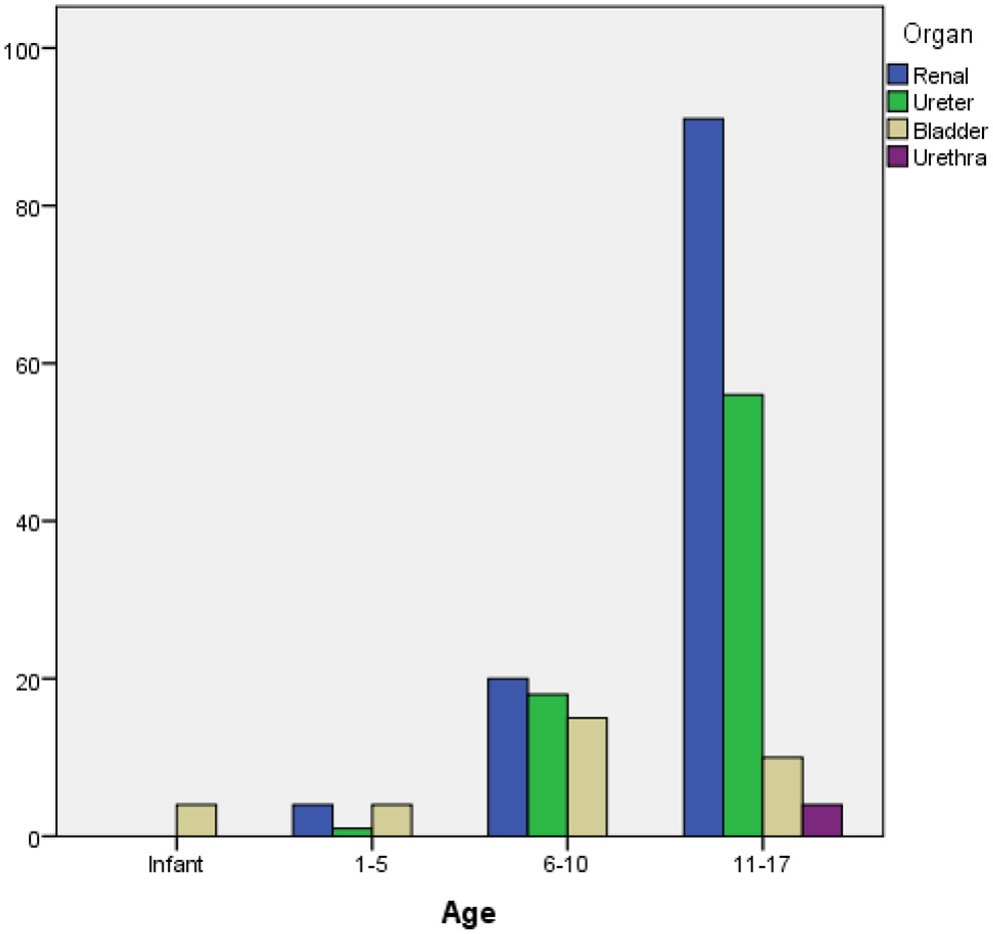
Distribution of stone locations based on age groups.

More than two-thirds of the patients (*n* = 161, 70.9%) were adolescents (i.e., aged between 11 and 17), while 23.3% were in the 6–10 age group (Table 1). There was a significant association between age group and stone location (*p* < 0.001) (Figure 1). As per our analysis, 80 and 74% of renal and ureteral stone cases were adolescents, while 70% of patients with bladder stones were younger than 10.

**Table 1:** Sociodemographic, radiological, and treatment data of the patients.

**Variables**	**Number of patients**	**Percentage (%)**
**Age groups**
Infant	4	1.8%
1–5	9	4%
6–10	53	23.3%
11–17	161	70.9%
**Gender**
Male	127	55.9%
Female	100	44.1%
**Stone location**
Kidney	115	50.7%
Ureter	75	33%
Bladder	33	14.5%
Urethra	4	1.8%
**Stone size**
<5 mm	15	6.6%
6–10 mm	53	23.3%
11–20 mm	96	42.3%
21–30 mm	37	16.3%
>30 mm	26	11.5%
**Grade of hydronephrosis**
No	38	16.7%
Grade 1	52	22.9%
Grade 2	69	30.4%
Grade 3	42	18.5%
Grade 4	26	11.4%
**Management of the patients**
Conservative	20	7.8%
URSL	40	18%
RIRS	30	13.2%
PCNL	31	14%
Pyelolithotomy	53	22.3%
Ureterolithotomy	15	6.6%
Cystolithotomy	36	15.9%
Nephrectomy	5	2.2%

The stones were located in the kidney in 50.7%, the ureter in 33%, and the bladder in 14.5% of our patients. The least common stone location was the urethra (1.8%).

**Figure 2:**
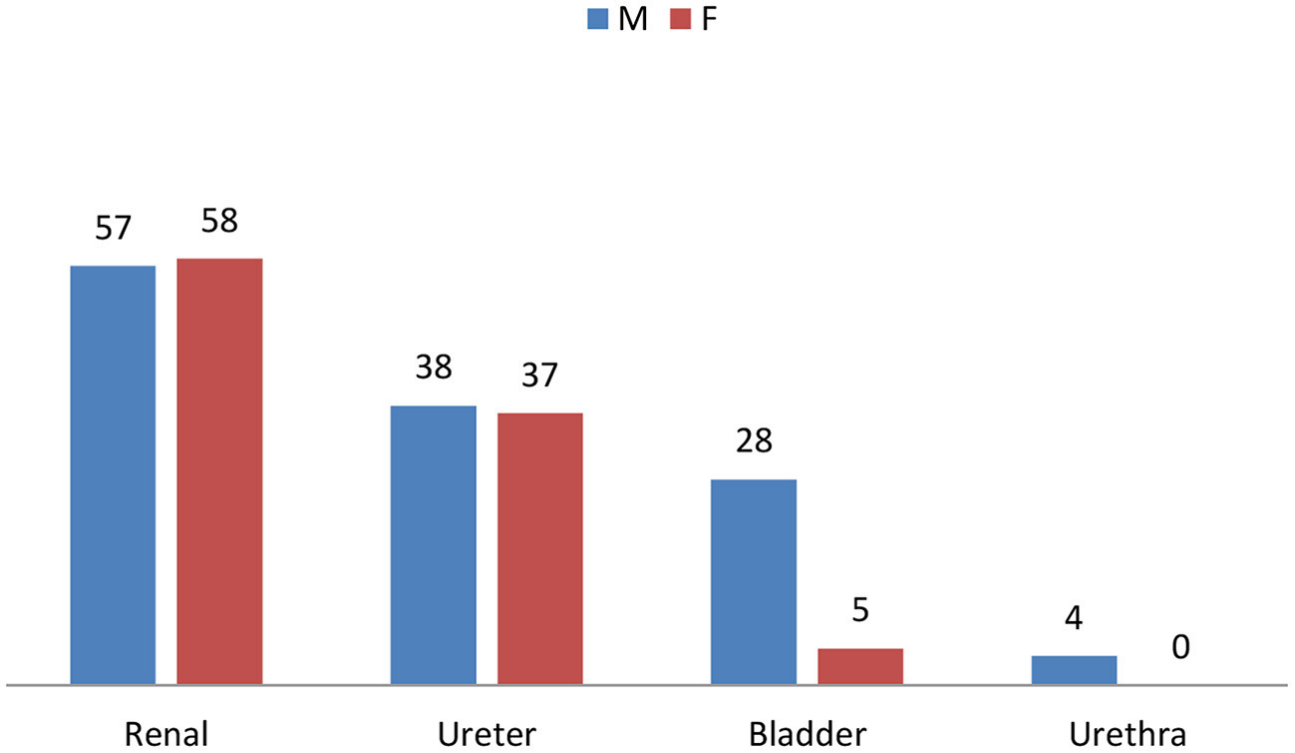
Stone locations based on gender distribution.

There was a statistically significant association between gender and stone location (*p* = 0.001). While 58% of the females had renal stones, 85% of bladder stone cases were males (Figure 2).

One-third of the cases had multiple stones; there was bilateral involvement in 12.3% and unilateral involvement in 70.5% of the cases. Distal ureteral stones accounted for the majority of ureteral stones (36%), followed by the ureterovesical junction (26.7%), proximal ureteral (24%), and mid-ureteral stones (13.3%).

**Figure 3:**
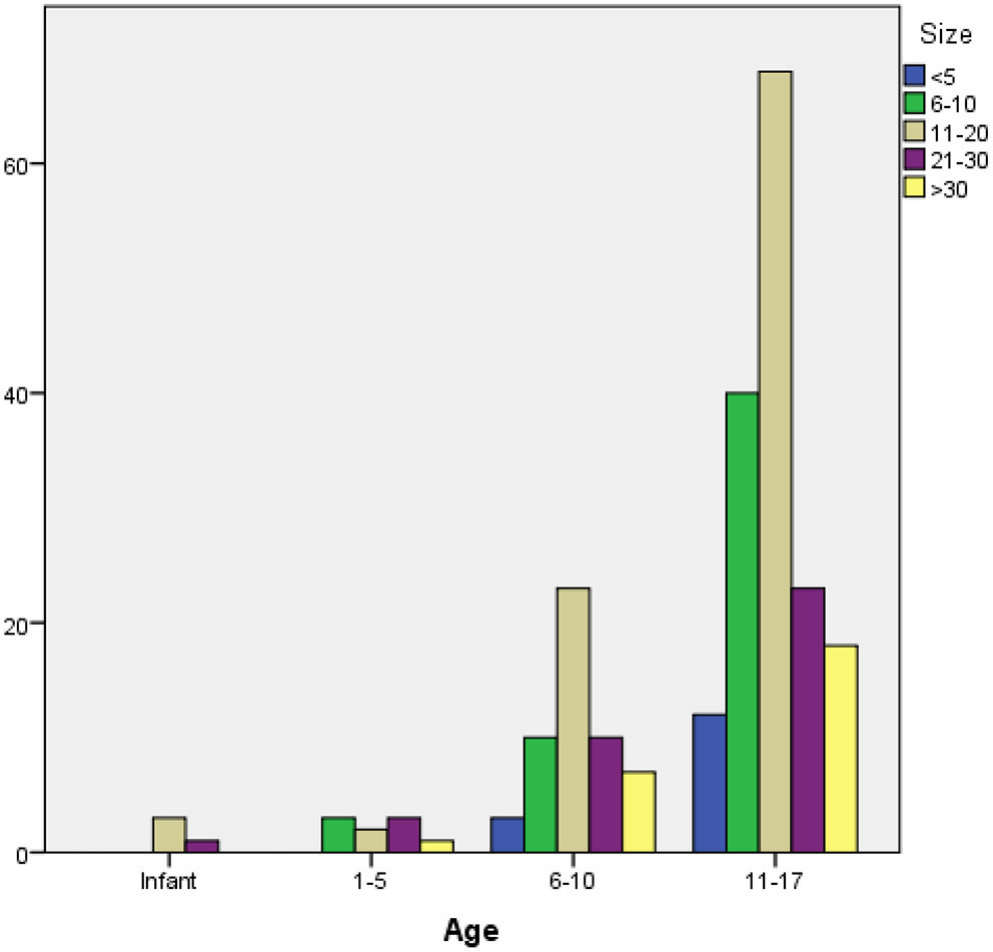
Stone sizes and age groups.

The mean stone size was 16.15 ± 5.6 [3–60]. Classification of the stones based on stone size revealed that most (42.3%) stones had a 10–20 mm diameter, while 23.3% were sized between 6 and 10 mm. Eight percent of the cases had staghorn stones. Renal stones had a significantly higher mean diameter than the stones at other locations (*p* < 0.001). The mean stone diameters were 17 ± 6.8, 14 ± 3.2, and 8.9 ± 4.5 mm in the kidney, bladder, and ureter stones. However, there was no significant association between age, gender, and stone size (*p* = 0.79, *p* = 0.166) (Figure 3).

In our cohort, the most common accompanying urological abnormalities were ectopic pelvic kidney (2.4%), horseshoe kidney (1.8%), and ureteropelvic junction obstruction (0.9%). In addition, most of the cases had grade 2 hydronephrosis (30.4%), followed by grade 1 (22.9%), grade 3 (18.5%), and grade 4 (11.4%).

Renal insufficiency secondary to obstructive uropathy was diagnosed in 5.3% (*n* = 12) of the cases. Bilateral involvement was present in all of these patients. Of note, 17.6% of the cases had various degrees of proteinuria (i.e., 7.75% <30 mg, 5.7% 30–300 mg, and 4.4% >300 mg). In addition, there were 3 cases with hyperuricemia and one case with hypercalcemia. Urinary tract infections were detected in 26% of the cohort.

Among 227 patients, 101 (44.5%) underwent minimally invasive procedures (MIP) including ureterorenoscopic lithotripsy (URSL) (*n* = 40, 18%), flexible ureteroscopy (FURS) (*n* = 30, 13.2%) and percutaneous nephrolithotomy (PCNL) (*n* = 31, 14%). Conversion to open surgery was needed in 6 patients. Open pyelolithotomy was the most common surgical procedure performed in the entire cohort (*n* = 53, 22.3%) (Table 1). Nephrectomy was needed in 5 patients due to non-functioning hydronephrotic kidney. These patients were diagnosed with non-functioning kidneys with IVU or CT-urography. Twenty (7.8%) patients were treated conservatively with medical expulsive therapy (MET).

Our review regarding complications revealed that 33 (14.9%) patients who underwent MIP needed an additional procedure such as double J stent insertion due to the inability to pass the ureteroscope, upward stone migration, or steinstrasse after lithotripsy. Three patients who underwent PCNL had intraoperative or postoperative bleeding, which necessitated a blood transfusion. In addition, two patients who underwent MIP developed urosepsis. On the other hand, 3.5% of the patients who went through open procedures developed surgical site infections. All of these cases were treated conservatively. No mortality was recorded in our study.

The mean hospital stay was 3.4 ± 1.6 [1–8] days. The length of hospital stay was significantly longer in patients who underwent open surgeries than in those who went through MIP (4.1 ± 1.5 vs. 1.7 ± 0.8 days, *p* < 0.001). The stone-free rate was 91.3% in the entire cohort (Table 2). Of note, this figure was significantly higher for open cases (99%) compared to the cases performed via a minimally invasive approach (83%) (*p* = 0.02).

**Table 2:** Stone-free rates following minimally invasive and open surgical procedures.

**Management**	**Stone-free rate**
Cystolithotomy	100%
Ureterolithotomy	100%
Pyelolithotomy	97%
Ureterorenoscopic lithotripsy	89%
Percutaneous nephrolithotomy	84%
Retrograde intrarenal surgery	78%

## Discussion

Since there are very few studies regarding PU reported from Africa, this disease remains poorly documented or underreported in this continent (13). A study from Egypt by Loutfi et al. reported 100 PU patients with a mean age of 5.8 years [14 months−12 years]. The stones were located in the upper urinary tract in 78%, the lower urinary tract in 19%, and both in 3%. They reported a PU rate of 12.6%. Another study reported from Tunisia included 133 children with a male to female ratio of 1.7:1 (14). This research had a study period of 12 years, and it revealed that upper urinary tract stones were most frequently detected (75%), while the rates of pediatric bladder stones were progressively increasing. In a retrospective study of 247 adult and pediatric patients from Ethiopia, the rate of PU was 6.4% (15). A small cross-sectional study of 21 pediatric patients from Cameroon revealed that bladder stones comprised 70% of PU cases (16). However, no previous research has been reported from Somalia on this subject. Therefore, we intended to evaluate the sociodemographic and radiological features, treatment, and outcome data of PU patients in Somalia.

Our study revealed that 83.7% of the cases had upper urinary tract stones, while 16.3% were diagnosed with lower urinary tract stones. This finding aligns with several previously published reports (13–15). However, in the study reported from Cameroon, the authors found a very high rate of bladder stones, and they ascribed this finding to insufficient diuresis and infections associated with malnutrition (16). It is widely accepted that bladder stones are common in Africa due to relatively higher malnutrition rates associated with diminished phosphorus consumption and vitamin A intake (17). Onal et al. reported that the rate of pediatric bladder stones was in the range of 1–5% in industrialized countries (17). In our study, the rate of bladder stones was 14.5%, which is a significantly higher rate than those reported by Onal et al.

It is known that the prevalence of PU varies between different countries due to the variations in the geographical and climatic zones, ethnicity, dietary habits, and socioeconomic conditions (9). In our study, we calculated the rate of PU as 8.1%; therefore, we suggest that PU is more common in Somalia than in many other countries (8, 15). The relatively higher rates of PU in Somalia can be due to the hot climate, improper water sanitation, and shortage of water resulting in low fluid intake, malnutrition, and genetic predisposition.

According to the previously published reports regarding PU, the rate of PU in the adolescent patient population increased to peak levels over the past 25 years, a phenomenon referred to as the “stone wave” (4). Our findings support this phenomenon; more than two-thirds of our patients were adolescents.

The gender distribution of pediatric urinary stone disease seems to differ with age; males have a higher prevalence of stone disease in the first 10 years of age, while there is a relatively higher female predominance in the second decade of life (18). Our study also noted this variation; 76% of our pediatric female patients were in their second decade of life (i.e., 11–17 years).

The EAU recommends US as the first initial imaging method since it is safe, readily available, and radiation-free (19, 20). In addition, it has a sensitivity and specificity of 61–93 and 95–100%, respectively. On the other hand, NCCT is used as the second-line imaging modality in this setting. Its sensitivity and specificity are both in the range of 96–100% (3). In our cohort, we had to proceed with NCCT in 79.2% of our patients to further delineate the stone location and diameter. In addition, we used NCCT during decision-making processes before performing PCNL.

In our study, 7.8% (*n* = 20) of the patients were treated conservatively by MET. Tamsulosin 0.2–0.4 mg/day or doxazosin 0.03/mg/kg/day was prescribed for MET. There are only a few studies in the literature regarding the use of MET as an off-label treatment in children with ureteral stones, and the results are controversial (21). It is known that URSL is increasingly used to treat pediatric ureteral stones worldwide (22, 23). This procedure has a 90% success rate as per the relevant literature (23). The RIRS is also an effective treatment modality for PU, with a success rate of 76–100% (24). Another surgical procedure used to treat urolithiasis is PCNL, and miniaturization of tract size has facilitated the use of PCNL in the pediatric patient population (25, 26). This procedure is safe and effective in experienced hands, with a success rate ranging between 70.1 and 97.3%. Open surgery is rarely used in developed countries; however, it is widely used in Sub-Saharan African countries like Somalia due to the limited availability of extracorporeal shock wave lithotripsy (ESWL), endourological equipment, and inadequate expertise. In line with this, 55.5% of all surgical procedures were open surgeries in our cohort. Our stone-free rate was 91.3% based on the evaluations performed 1 month after surgery. The stone-free rate was higher in patients who underwent open procedures than in those who went through MIP.

Although our study is the first study regarding the demographic data, radiological features, treatment, and outcomes of pediatric urolithiasis patients in Somalia, it has some limitations which need to be considered while evaluating its findings. First, it is a retrospective, single-center study. Second, it did not include stone analysis, metabolic analysis, and data regarding etiological investigations and long-term follow-up. Of note, stone analysis and metabolic analysis are not available in Somalia due to economic restrictions. Also, due to financial reasons, patients and their families cannot present for etiological investigations and periodic follow-up appointments.

## Conclusion

Despite the limitations mentioned above, we conclude that pediatric urolithiasis is common in Somalia as it is in other Sub-Saharan African countries. Considering the restricted endourology resources in Somalia, open surgery continues to be the primary treatment modality for children with urinary tract stone disease. However, minimally invasive approaches have been introduced over the last years. Since pediatric urolithiasis is associated with significant morbidity, including recurrence and renal insufficiency, immediate diagnosis and treatment are needed. Therefore, it should not be underestimated.

## Data Availability Statement

The original contributions presented in the study are included in the article/supplementary material, further inquiries can be directed to the corresponding author/s.

## Ethics Statement

Before commencing this study, approval was obtained from the Institutional Ethical Review Board of Mogadishu Somalia Turkish training and Research Hospital (MSTH-9006/21.02.2022). In addition, all study participants previously consented to using their medical and surgical data in the context of this study. Written informed consent to participate in this study was provided by the participants' legal guardian/next of kin.

## Author Contributions

AE, AM, and SC: study concept, design, interpretation, and drafting of the manuscript. All authors approved the final version of the manuscript.

## Conflict of Interest

The authors declare that the research was conducted in the absence of any commercial or financial relationships that could be construed as a potential conflict of interest.

## Publisher's Note

All claims expressed in this article are solely those of the authors and do not necessarily represent those of their affiliated organizations, or those of the publisher, the editors and the reviewers. Any product that may be evaluated in this article, or claim that may be made by its manufacturer, is not guaranteed or endorsed by the publisher.
